# Machine learning prediction model of acute kidney injury after percutaneous coronary intervention

**DOI:** 10.1038/s41598-021-04372-8

**Published:** 2022-01-14

**Authors:** Toshiki Kuno, Takahisa Mikami, Yuki Sahashi, Yohei Numasawa, Masahiro Suzuki, Shigetaka Noma, Keiichi Fukuda, Shun Kohsaka

**Affiliations:** 1 Division of Cardiology, Montefiore Medical Center, Albert Einstein College of Medicine, 111 East 210th St, Bronx, NY 10467-2401 USA; 2 Department of Neurology, Tufts Medical Center, Boston, MA USA; 3 Department of Cardiovascular Medicine, Gifu Heart Center, Gifu, Japan; 4 Department of Cardiology, Gifu University Graduate School of Medicine, Gifu, Japan; 5 Department of Health Data Science, Graduate School of Data Science, Yokohama City University, Yokohama, Japan; 6 Department of Cardiology, Japanese Red Cross Ashikaga Hospital, Ashikaga, Japan; 7 Department of Cardiology, Saitama National Hospital, Wako, Japan; 8 Department of Cardiology, Saiseikai Utsunomiya Hospital, Utsunomiya, Japan; 9 Department of Cardiology, Keio University School of Medicine, Tokyo, Japan

**Keywords:** Cardiology, Interventional cardiology

## Abstract

Acute kidney injury (AKI) after percutaneous coronary intervention (PCI) is associated with a significant risk of morbidity and mortality. The traditional risk model provided by the National Cardiovascular Data Registry (NCDR) is useful for predicting the preprocedural risk of AKI, although the scoring system requires a number of clinical contents. We sought to examine whether machine learning (ML) techniques could predict AKI with fewer NCDR-AKI risk model variables within a comparable PCI database in Japan. We evaluated 19,222 consecutive patients undergoing PCI between 2008 and 2019 in a Japanese multicenter registry. AKI was defined as an absolute or a relative increase in serum creatinine of 0.3 mg/dL or 50%. The data were split into training (N = 16,644; 2008–2017) and testing datasets (N = 2578; 2017–2019). The area under the curve (AUC) was calculated using the light gradient boosting model (GBM) with selected variables by Lasso and SHapley Additive exPlanations (SHAP) methods among 12 traditional variables, excluding the use of an intra-aortic balloon pump, since its use was considered operator-dependent. The incidence of AKI was 9.4% in the cohort. Lasso and SHAP methods demonstrated that seven variables (age, eGFR, preprocedural hemoglobin, ST-elevation myocardial infarction, non-ST-elevation myocardial infarction/unstable angina, heart failure symptoms, and cardiogenic shock) were pertinent. AUC calculated by the light GBM with seven variables had a performance similar to that of the conventional logistic regression prediction model that included 12 variables (light GBM, AUC [training/testing datasets]: 0.779/0.772; logistic regression, AUC [training/testing datasets]: 0.797/0.755). The AKI risk model after PCI using ML enabled adequate risk quantification with fewer variables. ML techniques may aid in enhancing the international use of validated risk models.

## Introduction

Acute kidney injury (AKI) is a common non-cardiovascular complication of percutaneous coronary intervention (PCI) and is associated with a significantly increased risk of morbidity and mortality during and after hospitalization^1–3^. An use of established prediction model to estimate the risk of AKI after PCI may improve clinical decisions to avoid AKI with adequate hydration and reduction of contrast volume^2,4^. Numerous AKI prediction models for PCI have been investigated in a variety of countries and regions^5–8^, albeit only a few of these models have been externally and internationally validated. The National Cardiovascular Data Registry (NCDR)-AKI risk scoring system is widely utilized and has been cross-validated in the Japanese registry internationally despite the differences in patient characteristics, including race^9–11^. Briefly, Tsai et al. developed the NCDR-AKI risk model in 2014 through a logistic regression model with 12 preprocedural variables^9^, splitting the data into 70% of the derivation cohort and 30% of the validation cohort with C-statistics of 0.72, and 0.71, respectively. This model was shown to be the preferable prediction model compared to other traditional AKI risk models^12^.

However, their traditional approach included transforming continuous variables into categorical variables when calculating risk scores. They also selected 12 variables via traditional stepwise selection, and further simplification of the model (e.g. reducing the number of variables) was not feasible since it likely reduces the overall predictability of the model. Recently developed machine learning (ML) methods may be able to enhance the performance of risk prediction. They allow nonlinear associations and are better suited for extracting additional information from continuous variables^13,14^. To date, AKI prediction models that limit covariates with ML, especially the light gradient boosting model (GBM), have been scarcely studied. The light GBM is conceived to reduce calculation time, which attracts attention because it might be suitable for the creation of prediction calculators on the website^15^. In addition, it also allows missing values for a prediction model, which is more advantageous than a conventional logistic regression model.

We sought to investigate whether ML-derived prediction models could predict AKI adequately or enhance risk predictions with fewer variables selected via least absolute shrinkage and selection operator (Lasso) and SHapely Additive exPlanations (SHAP) using covariates within a comparable PCI database in Japan. The AKI risk model using ML could be widely implemented if the number of variables could be successfully reduced without decreasing the model’s predictability. Further, ML may serve to be a useful tool when considering the application of risk model to patients in different geographical regions.

## Methods

The Japanese Cardiovascular Database-Keio Inter-hospital Cardiovascular Studies (JCD-KiCS) registry is a large prospective multicenter cohort study that collects consecutive clinical background and outcome data on patients undergoing PCI. Prior reports have clarified the data collection procedures and audit processes for the JCD-KiCS^10,16–18^. Before the launch of the JCD, information on the study objectives, its social significance, and an abstract were provided to register this clinical trial with the University Hospital Medical Information Network. This Network is recognized by the International Committee of Medical Journal Editors as an acceptable registry, according to a statement issued in September 2004 (UMIN R000005598). Written informed consent was obtained from each patient before inclusion in the study.

The data entered were assessed for completeness and internal consistency. Quality assurance was achieved through automatic system validation, reporting of data completeness, and education of dedicated clinical research coordinators specifically trained for this PCI registry. The senior study coordinator and extensive on-site auditing by the investigator (S.K.) ensured appropriate registration of each patient. The present study was approved by the institutional review board Committee of Keio University (Reference Number: 20080073) and was conducted in accordance with the principles of the Declaration of Helsinki.

In addition, the JCD-KiCS previously validated the NCDR-AKI risk model^10^. The majority of the clinical variables included in the JCD-KiCS were defined according to the NCDR, sponsored by the American College of Cardiology^11^. In the JCD-KiCS registry, 15 hospitals from Tokyo, Tochigi, Saitama, Chiba, and Kanagawa Prefectures in the Kanto area were included^4^. Twelve of the 15 participating hospitals were large tertiary-care referral centers (≥ 200 beds), while three were mid-sized satellite hospitals (< 200 beds). The average annual case volume was approximately 228 between 2011 and 2013 at these 15 hospitals. After 2013, only four high-volume centers were included (the average case volume was 479).

**Figure 1:**
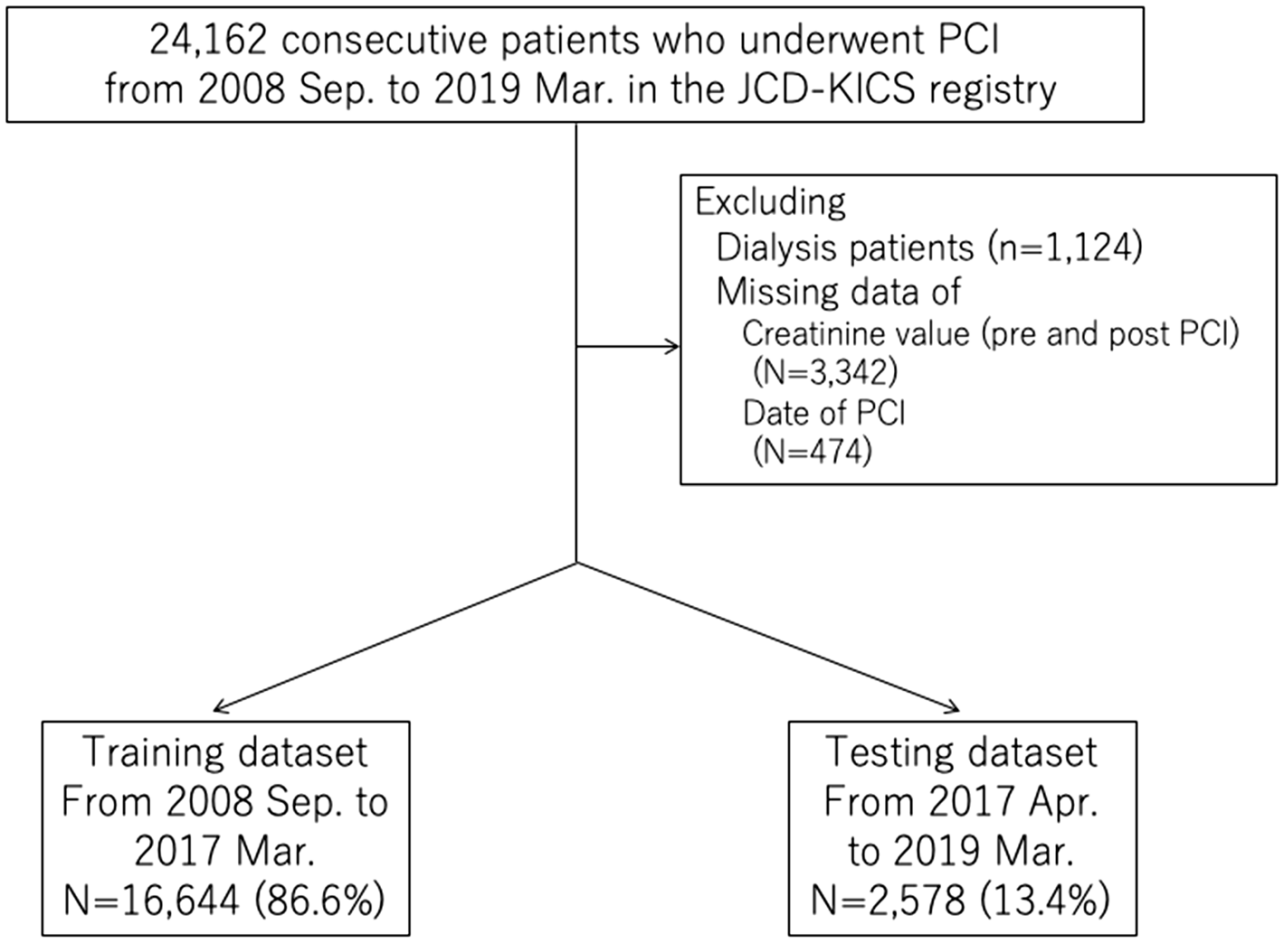
Patients’ flow chart. PCI: percutaneous coronary intervention, JCD-KiCS: Japanese Cardiovascular Database-Keio Inter-hospital Cardiovascular Studies.

Patients on chronic dialysis (N = 1124) and those with missing data on AKI after PCI (N = 3342) were excluded. In addition, those with missing data on the date when PCI was performed (N = 474) were also excluded because we split the data into training and testing datasets based on the date of PCI (Fig. 1). In this retrospective study, we evaluated 19,222 consecutive patients undergoing PCI between September 2008 and March 2019 using the JCD-KiCS registry. AKI was defined as an absolute increase of 0.3 mg/dL or a relative increase of 50% in serum creatinine^10^. New induction of dialysis was also considered as AKI given its severity^12^. In the JCD-KiCS registry, the post-procedural creatinine value was defined as the highest value within 30 days after the index procedure. Congruent with the NCDR definition, if more than one post-procedural creatinine level was measured, the highest value was used to determine the incidence of AKI. This timing of peak creatinine was congruent with that reported in previous studies^10^.

PCI-related complications were defined as a composite endpoint that included severe flow-limiting coronary dissection or coronary perforation, myocardial infarction after PCI, cardiogenic shock or heart failure, cerebral bleeding or stroke, and other bleeding complications. Bleeding complications were defined as those requiring transfusion, prolonging hospital stay, and/or causing a decrease in hemoglobin of > 3.0 g/dl^19^. When present, bleeding complications were classified by the site of bleeding, as follows: puncture-site bleeding, including external bleeding; or a hematoma > 10 cm for femoral, > 5 cm for brachial, or > 2 cm for radial access sites; retroperitoneal bleeding; gastrointestinal bleeding; genitourinary bleeding; or other types of bleeding. This definition of bleeding complications was consistent with the definition of grade 3 (A to C) bleeding from the Bleeding Academic Research Consortium^20^. Our definitions of complications were consistent with the NCDR CathPCI registry, and additional information on data elements and definitions is available on their website (http://www.ncdr.com/webncdr/cathpci/).

Continuous variables were expressed as mean and standard deviation or median (interquartile range), as appropriate for the data distribution, with categorical variables expressed as percentages. The change from baseline in continuous variables was evaluated using Student’s *t*-test or the Mann–Whitney U test, as appropriate for the data distribution, with the chi-squared or Fisher’s exact test used for categorical variables.

To construct the ML model, we initially identified the important variables associated with AKI after PCI using Lasso through logistic regression. Lasso selects variables by shrinking the coefficients of less important variables from logistic regression to zero^21^. The twelve variables used for the NCDR-AKI risk model (defined as the NCDR variables) were included in the Lasso model^9^: age (< 50, 50–59, 60–69, 70–79, 80–89, and > 90), chronic kidney disease (estimated glomerular filtration rate [eGFR] [mL/min./1.73 m^2^], 60<, 45–60, 30–45, < 30), previous heart failure, diabetes mellitus, cerebrovascular disease, heart failure at admission, cardiogenic shock (CS) at admission, cardiopulmonary arrest (CPA) at admission, use of intra-aortic balloon pump (IABP), ST-elevation myocardial infarction, non-ST-elevation myocardial infarction/unstable angina, and preprocedural hemoglobin (< 10 g/dL). In addition, we constructed the SHAP approach to select the important variables with the light GBM using the 12 NCDR variables. This approach explains the models at the level of individual patients based on the sum of the numeric computed credit (SHAP) values of each feature^22,23^. Categorical variables (age, eGFR, and preprocedural hemoglobin) were entered as continuous variables in the Lasso and SHAP models.

After the selection of important variables, the data were split into training and testing datasets: before March 31, 2017 (N = 16,644, 86.6%) and after April 1, 2017 (N = 2578, 13.4%), respectively, to validate the data in recent years, which is considered an effective model for the current practice (Fig. 1, Supplemental Table 1). Subsequently, a light GBM model using a stratified K-fold cross-validation method was applied to the training dataset (K = 5)^24^. The light GBM was designed to be accurate, efficient, and fast, which are advantages in handling large-scale data^22^. In comparison to the logistic regression model, the light GBM used “NaN” to represent missing values and were dealt with separately from zero, as missing values were interpreted as containing information^22^. Hyperparameter optimization was performed using an implementation called “Optuna”^25^. Categorical variables were entered as continuous variables to leverage ML techniques^15,24^. We used the area under the curve (AUC) to evaluate the different models, and the AUC was calculated for the training and testing datasets. Furthermore, we analyzed two versions of light GBM models, with IABP, since the discretion of its use was considered operator-dependent, and the benefit of IABP has been considered reduced over the past years during this study period. Additionally, new mechanical circulatory support devices such as Impella (Abiomed, MA, USA) are emerging, but their benefits remain unproven^26,27^. Although the NCDR-AKI risk model included IABP^9^, we also selected the pertinent variables with SHAP and Lasso without IABP and analyzed the AKI risk model using the light GBM without IABP.

**Table 1:** Baseline characteristics of all patients.

	Patients without AKI N = 17,422 (%)	Patients with AKI N = 1800 (%)	*P* value
Age	68.2 (11.2)	71.1 (12.0)	< 0.001
Male	13101 (79.5)	1300 (76.8)	0.011
Creatinine value (mg/dl)	0.99 ± 0.85	1.38 ± 1.29	< 0.001
eGFR (mL/min./1.73 m^2^)	64.6 ± 19.5	54.7 ± 26.3	< 0.001
Baseline Hemoglobin (g/dl)	13.5 ± 2.0	12.8 ± 2.6	< 0.001
Previous myocardial infarction	3863 (23.5)	293 (17.4)	< 0.001
Previous heart failure	1386 (8.4)	243 (14.4)	< 0.001
Diabetes mellitus	6708 (41.0)	762 (45.6)	< 0.001
Cerebrovascular disease	1372 (8.3)	209 (12.4)	< 0.001
Peripheral artery disease	1335 (8.1)	173 (10.3)	0.002
Chronic lung disease	532 (3.2)	63 (3.7)	0.26
Hypertension	12,083 (73.5)	1323 (78.4)	< 0.001
Dyslipidemia	10,849 (66.1)	968 (57.5)	< 0.001
Previous PCI	6291 (38.1)	348 (20.6)	< 0.001
Previous coronary bypass	780 (4.7)	85 (5.0)	0.51
Heart failure on admission	1672 (9.6)	517 (28.7)	< 0.001
Cardiogenic shock on admission	548 (3.1)	327 (18.2)	< 0.001
Cardiopulmonary arrest on admission	348 (2.0)	217 (12.1)	< 0.001
Radial artery approach	9310 (53.6)	577 (32.1)	< 0.001
Intra-aortic balloon pump	906 (5.2)	519 (28.8)	< 0.001
ST-elevation myocardial infarction	3673 (21.2)	915 (51.2)	< 0.001
UA/NSTEMI	3989 (23.0)	410 (22.9)	0.98

±: SD, AKI: acute kidney injury, eGFR: estimated glomerular filtration rate, PCI: percutaneous coronary intervention UA/NSTEMI: unstable angina/non ST-elevation myocardial infarction.

A multivariate logistic regression model was constructed to predict the incidence of AKI using the 12 NCDR variables. The training and testing datasets were split as well, and the AUCs calculated in training using stratified K-fold cross-validation method were applied to the training dataset (K = 5) and testing datasets. We used a grid search strategy to identify the best tuning hyperparameters^28^. We also used the Standard Scaler to improve the predictability^29^. We performed an imputation for the missing data using the library of IterativeImputer in Python for logistic regression models because a logistic regression model does not allow missing values. AUCs were used to evaluate the different models. As a sensitivity analysis, we analyzed categorical variables instead of continuous variables for light GBM models in concordance with the NCDR-AKI risk model.

All statistical calculations and analyses were performed using R (version 3.6.2, R Foundation for Statistical Computing, Vienna, Austria) and Python 3.7 (Python Software Foundation Delaware, USA). Statistical significance was set at *P* < 0.05. Finally, we created a web-based calculator to predict the risk of AKI after PCI.

## Results

The patients’ mean age was 68.5 ± 11.3 years; the total AKI incidence was 9.4% (N = 1800). The baseline characteristics and in-hospital outcomes of patients with AKI versus those without AKI are shown in Tables 1 and 2. The patients with AKI had a significantly higher incidence of acute presentations, such as heart failure at admission, CS, cardiopulmonary arrest, ST-elevation myocardial infarction, and use of IABP (Table 1). Moreover, patients with AKI had significantly higher in-hospital mortality, transfusion, and procedural complications, including bleeding complications (Table 2).

**Table 2:** in-hospital mortality and complications.

	Patients without AKI N = 17,422 (%)	Patients with AKI N = 1800 (%)	*P* value
In-hospital mortality	145 (0.8)	275 (15.3)	< 0.001
All complications	1173 (6.8)	630 (35.3)	< 0.001
Coronary Dissection	134 (0.8)	34 (1.9)	< 0.001
Coronary Perforation	121 (0.7)	32 (1.8)	< 0.001
Myocardial infarction	228 (1.3)	64 (3.6)	< 0.001
Cardiogenic shock	186 (1.1)	178 (9.9)	< 0.001
Heart failure	188 (1.1)	181 (10.1)	< 0.001
Cerebral infarction	48 (0.3)	36 (2.0)	< 0.001
Intracranial hemorrhage	6 (0.03)	13 (0.7)	< 0.001
Cardiac tamponade	27 (0.2)	36 (2.0)	< 0.001
Hemodialysis	0 (0.0)	210 (11.7)	< 0.001
Transfusion	274 (1.6)	264 (14.7)	< 0.001
Bleeding all	373 (2.1)	244 (13.6)	< 0.001
Puncture site bleeding	104 (0.6)	62 (3.4)	< 0.001
Puncture site hematoma	107 (0.6)	31 (1.7)	< 0.001
Peritoneal bleeding	14 (0.1)	10 (0.6)	< 0.001
Gastrointestinal bleeding	31 (0.2)	43 (2.4)	< 0.001
Genitourinary bleeding	12 (0.1)	10 (0.6)	< 0.001
Other bleeding	141 (0.8)	129 (7.2)	< 0.001

AKI: Acute kidney injury.

**Figure 2:**
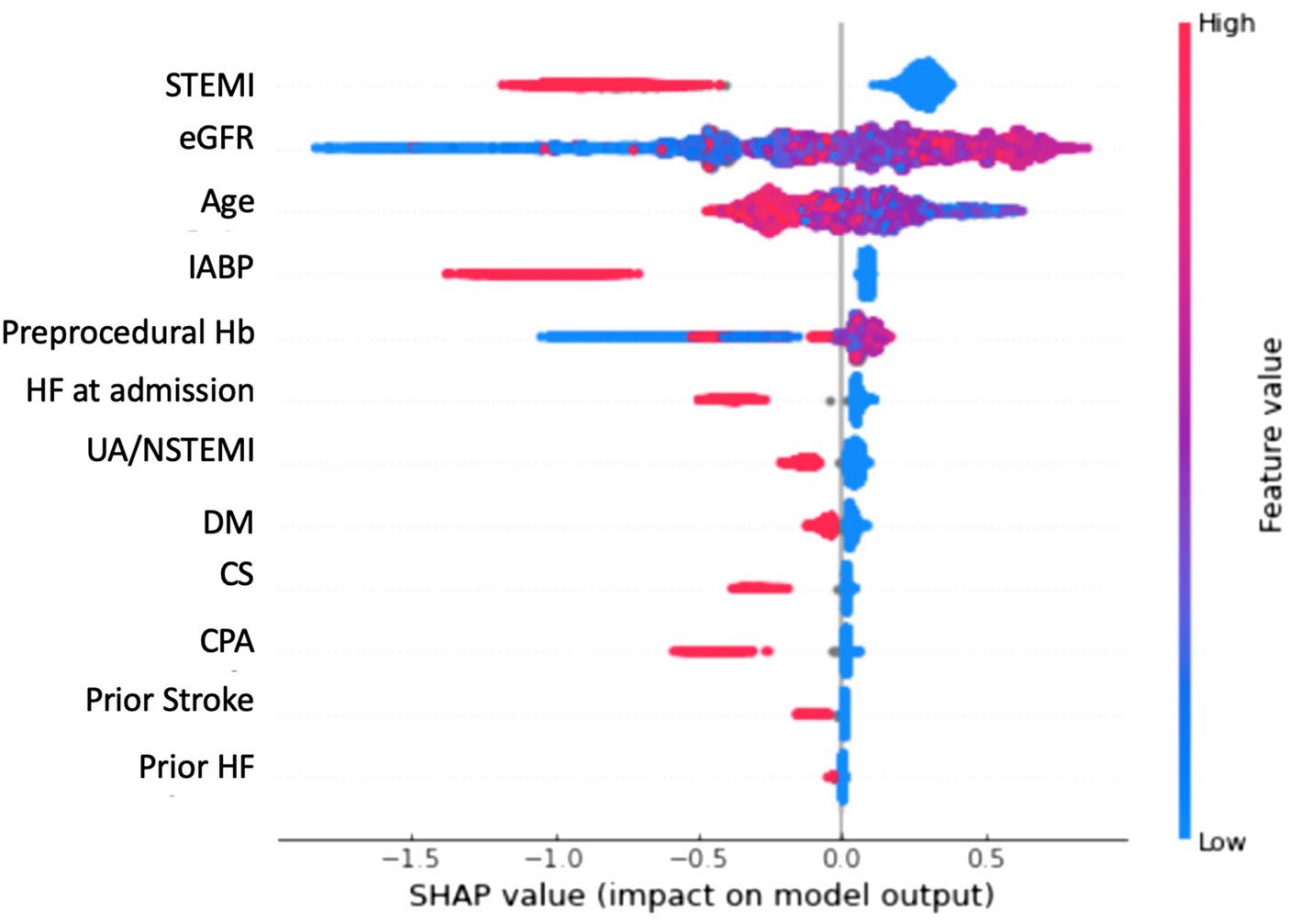
SHAP model to estimate important variables with the light gradient boosting model using the 12 NCDR variables. The features are sorted in descending order by Shapley values. Abbreviations: CS: cardiogenic shock; CPA: cardiopulmonary arrest; DM: diabetes mellitus, Hb: hemoglobin; HF: heart failure, IABP: intra-aortic balloon pump; STEMI: ST-elevation myocardial infarction; UA/NSTEMI: unstable angina/non-ST elevation myocardial infarction.

In the selection of pertinent variables, the Lasso method demonstrated that age, eGFR, preprocedural hemoglobin, ST-elevation myocardial infarction, non-ST-elevation myocardial infarction/unstable angina, heart failure symptoms at admission, use of IABP, and CS were important for predicting the risk of AKI after PCI. SHAP also demonstrated that these variables, except for CS, were also important (Fig. 2). After removing IABP from the 12 NCDR variables, the Lasso method demonstrated that age, eGFR, preprocedural hemoglobin, ST-elevation myocardial infarction, non-ST-elevation myocardial infarction/unstable angina, heart failure symptoms at admission, and CS and CPA were important predictors of the risk of AKI after PCI. SHAP also demonstrated that these features, except for CPA, were also important.

According to SHAP, we selected age, eGFR, preprocedural hemoglobin, ST-elevation myocardial infarction, non-ST-elevation myocardial infarction/unstable angina, and heart failure symptoms at admission as six basic variables. We subsequently added IABP or CS to these six variables. We also analyzed six variables + IABP + CS, or six variables + CS + CPA with the light GBM, according to the variables selected using the Lasso method.

**Figure 3:**
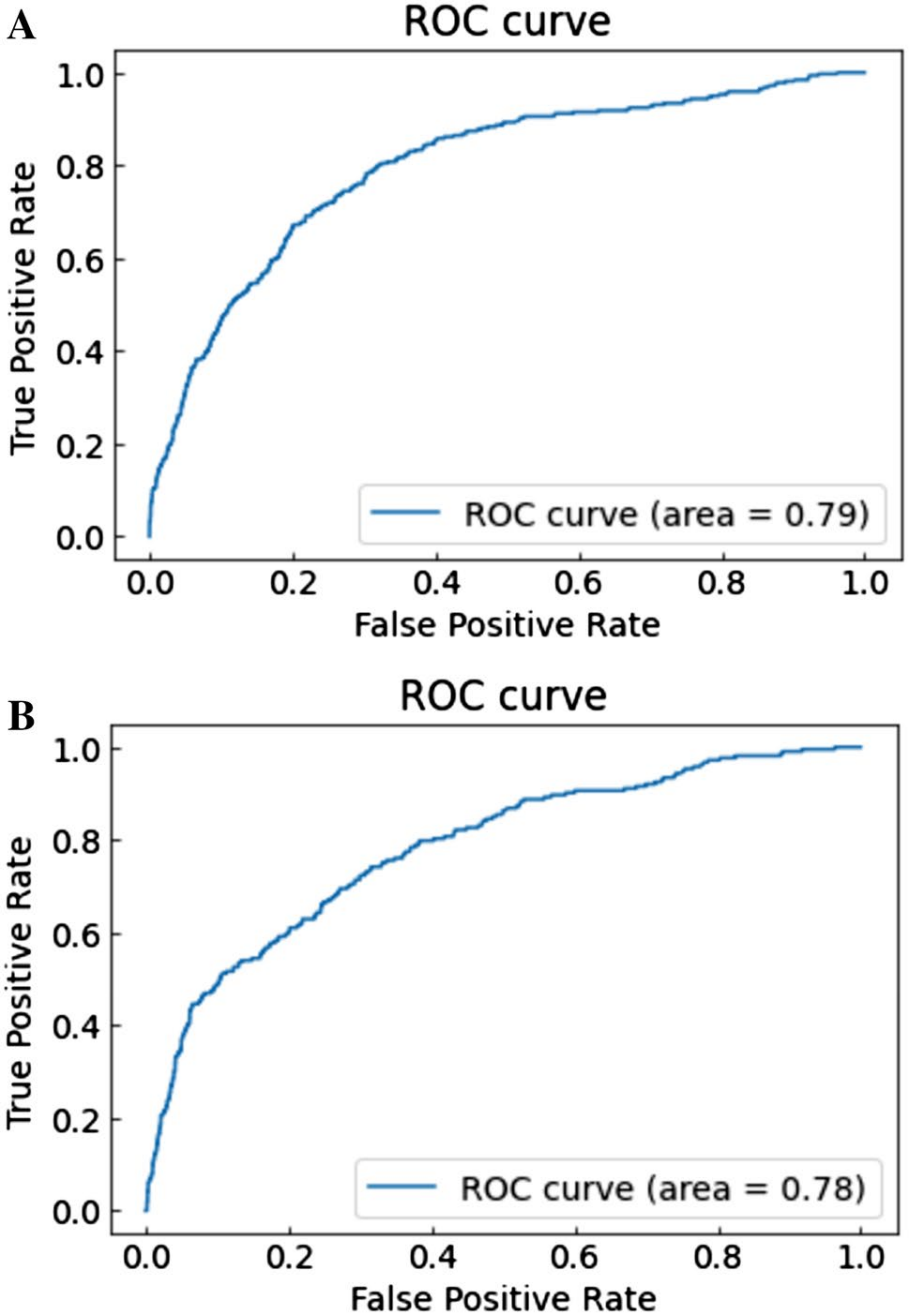
(**A**, **B**) ROC curve with training datasets using the light GBM with age, eGFR, preprocedural hemoglobin, ST-elevation myocardial infarction, non-ST elevation myocardial infarction/unstable angina, heart failure symptoms at admission and intra-aortic balloon pump. Abbreviations: GFR, glomerular filtration rate; GBM, gradient boosting model; ROC, receiver operating characteristic.

**Figure 4:**
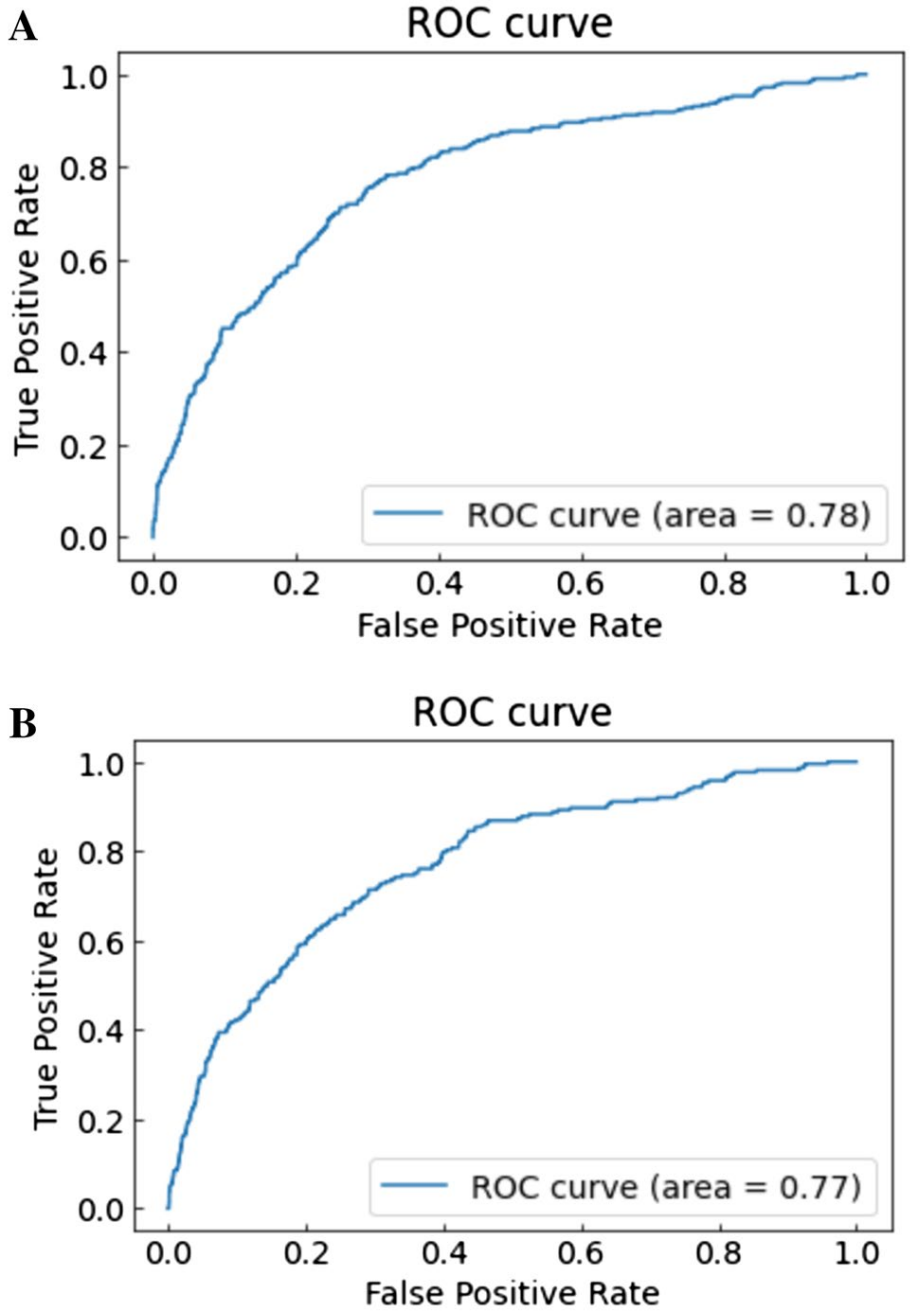
(**A**) ROC curve with training datasets using the light GBM with age, eGFR, preprocedural hemoglobin, ST-elevation myocardial infarction, non-ST elevation myocardial infarction/unstable angina, heart failure symptoms at admission and cardiogenic shock. (**B**) ROC curve with testing datasets using the light GBM age, eGFR, preprocedural hemoglobin, ST-elevation myocardial infarction, non-ST elevation myocardial infarction/unstable angina, heart failure symptoms at admission, and cardiogenic shock. Abbreviations: GFR: glomerular filtration rate, ROC: receiver operating characteristic.

**Figure 5:**
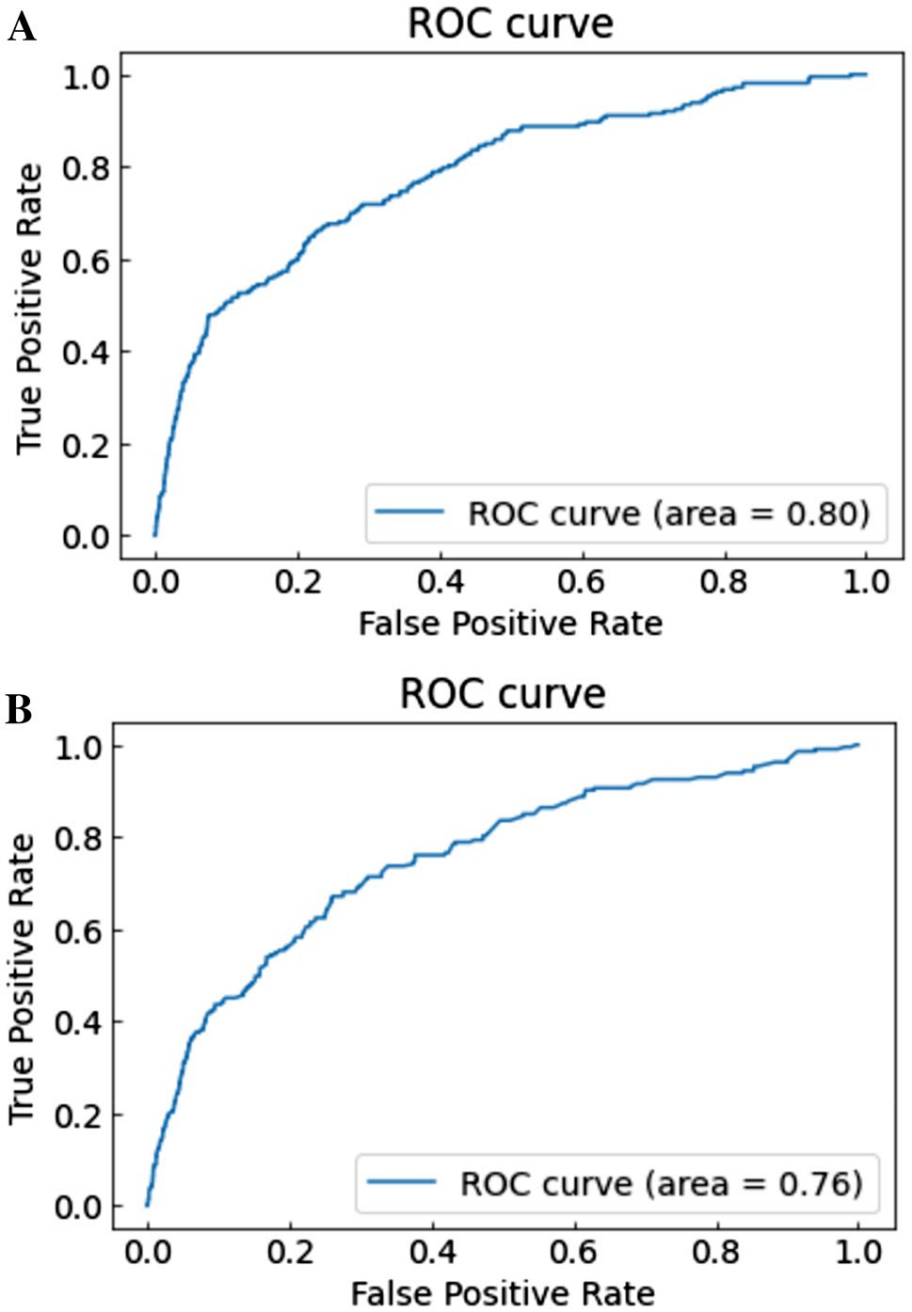
(**A**) ROC curve with training datasets using the logistic regression model with the 12 NCDR variables. (**B**) ROC curve with testing datasets using the logistic regression model with the 12 NCDR variables. Abbreviations: ROC: receiver operating characteristic.

Notably, the AUC calculated by the light GBM with six basic variables with IABP or CS had a performance similar to that of the conventional logistic regression prediction model that included 12 variables (light GBM by six variables + IABP, AUC [training/testing datasets]: 0.790/0.784 (receiver operating characteristic (ROC) curves: Fig. 3A,B); light GBM by six variables + CS, AUC [training/testing datasets]: 0.779/0.772 (ROC curves: Fig. 4A,B); logistic regression, AUC [training/testing datasets]: 0.797/0.755 (ROC curves: Fig. 5A,B). The results of the other versions of the selected variables are presented in Table 3. As a sensitivity analysis, we used categorical variables instead of continuous variables for the light GBM, which revealed low AUC scores in the testing datasets (Table 3).

**Table 3:** Area under the curve in different models.

Model	Continuous or categorical	IABP	CS	CPA	AUC (training)	AUC (testing)
Light GBM	Continuous	+	−	−	0.790	0.784
	Continuous	−	+	−	0.779	0.772
	Continuous	+	+	−	0.79	0.783
	Continuous	−	+	+	0.783	0.775
	Categorical	+	−	−	0.795	0.755
	Categorical	−	+	−	0.786	0.745
	Categorical	+	+	−	0.796	0.759
	Categorical	−	+	+	0.789	0.753
Logistic regression	Categorial	+	+	+	0.797	0.755

AUC, Area under the curve; CS, cardiogenic shock; CPA, cardiopulmonary arrest; GBM, gradient boosting model; IABP, intra-aortic balloon pump.

**Figure 6:**
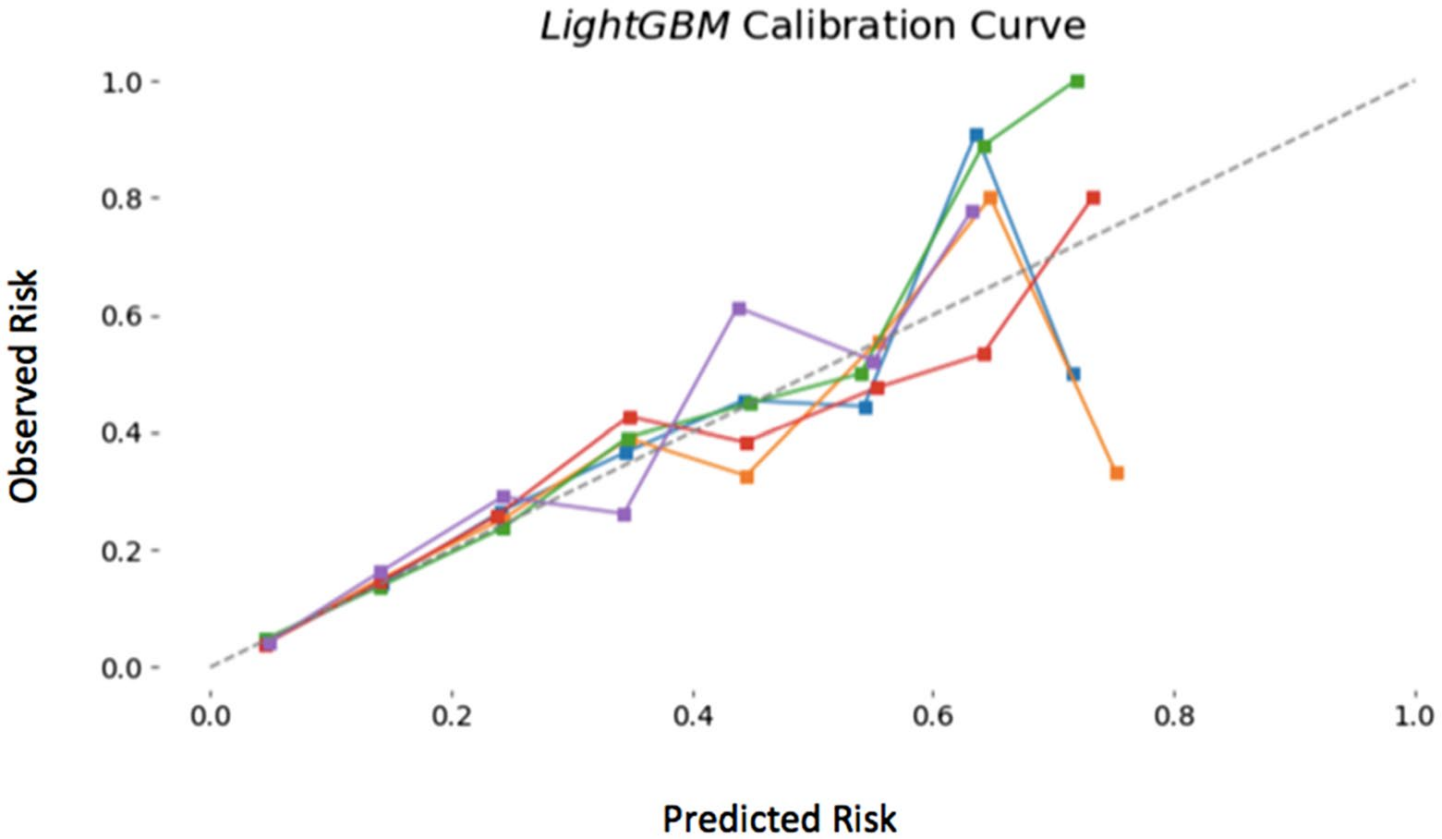
Calibration plot using the light GBM with age, eGFR, preprocedural hemoglobin, ST-elevation myocardial infarction, non-ST elevation myocardial infarction/unstable angina, heart failure symptoms at admission, and cardiogenic shock. Abbreviations: GBM: gradient boosting model; eGFR: estimated glomerular filtration rate.

**Figure 7:**
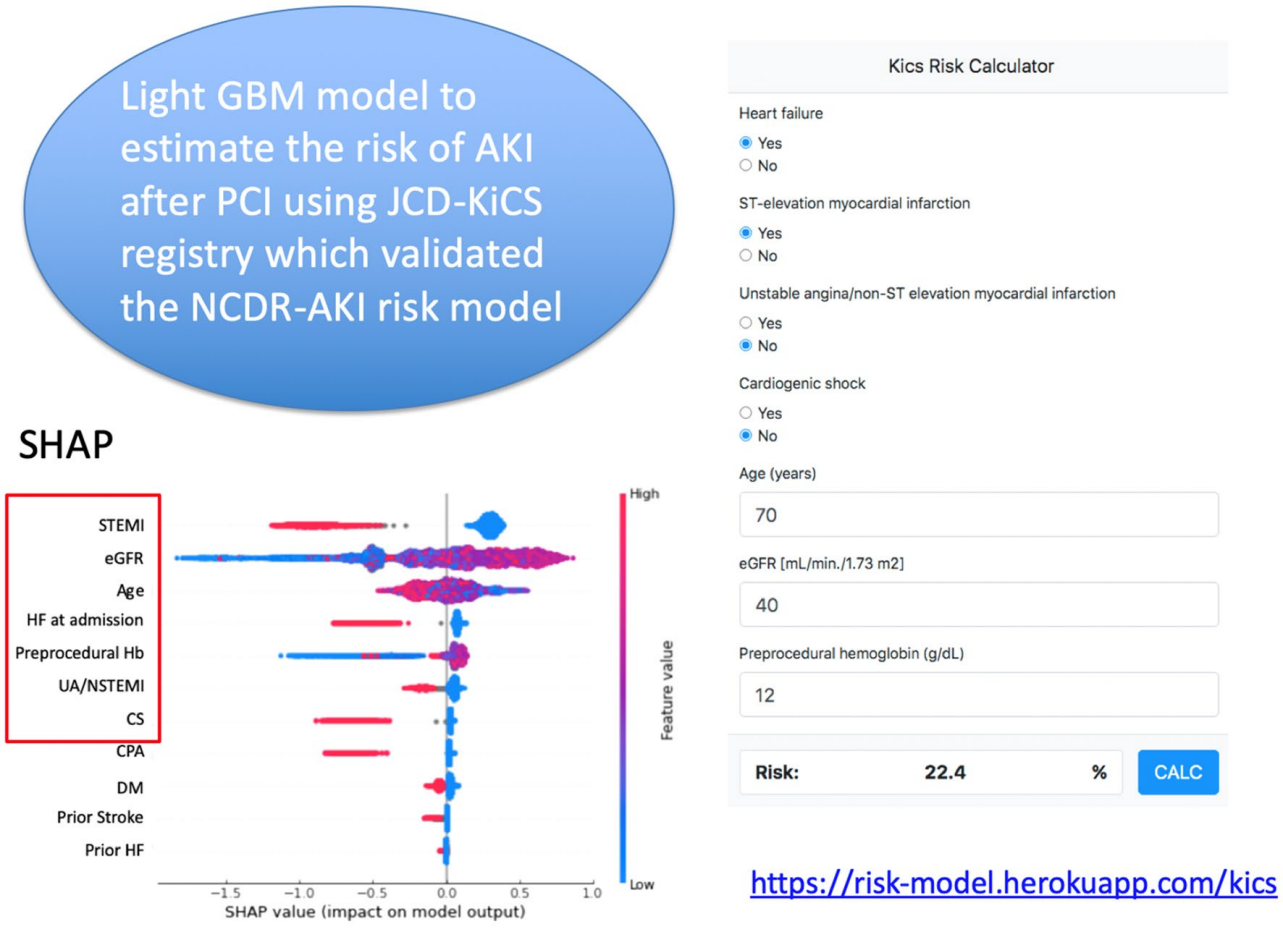
(Left) SHAP model to estimate important variables with the light gradient boosting model using the 12 NCDR variables except intra-aortic balloon pump. The features are sorted in descending order by Shapley values. Abbreviations: AKI: acute kidney injury; CS: cardiogenic shock; CPA: cardiopulmonary arrest; DM: diabetes mellitus; GBM: gradient boosting model; Hb: hemoglobin, HF: heart failure, IABP: intra-aortic balloon pump; JCD-KiCS: Japanese Cardiovascular Database-Keio Inter-hospital Cardiovascular Studies; NCDR: National Cardiovascular Data Registry; STEMI: ST-elevation myocardial infarction; UA/NSTEMI: unstable angina/non-ST elevation myocardial infarction. (Right) Kics risk calculator (https://risk-model.herokuapp.com/kics).

Figure 6 shows the calibration plots with the light GBM using the seven variables without IABP (including CS but not CPA), which demonstrates concordance between the probability predicted by the light GBM and the probability actually observed. The calculator on the website created with this model is at the website for clinical use (https://risk-model.herokuapp.com/kics). The sample screen is shown in Fig. 7.

## Discussion

The salient findings of our study are as follows (Fig. 6): (1) seven important variables (with IABP or CS) among the 12 NCDR variables were found to predict AKI after PCI, and (2) ML enabled adequate quantification of its risk with fewer clinical variables. (3) Using categorical variables in the conventional NCDR-AKI risk model may decrease the predictability in the testing dataset. ML techniques may aid in better selecting high-risk AKI patients, and in promoting the international use of traditional risk prediction models.

The traditional scoring system using the NCDR-AKI risk model is an effective in estimating the risk of AKI after PCI^9,10^. We used the definition of AKI as serum creatinine by ≥ 0.3 mg/dl or an increase to ≥ 50% since this definition is the most optimal to predict short-term and long-term adverse mortality as well as the NCDR-AKI risk model^9,12,30,31^. Additionally, we did not include contrast volume according to the NCDR-AKI risk model because the causes of AKI are multifactorial such as contrast or hemodynamically instability and pre-operative assessment the risk of AKI is paramount to decrease the amount of contrast volume. The enhanced ML risk prediction has been shown to enhance risk predictions even in the patients with the lowest and highest risks. However, these traditional risk scoring system requires, at most, 25 clinical variables^13^. Although these risk models are relatively effective in risk predictions, a number of variables needs to be entered and could be a burden for clinicians especially emergent cases such as ST elevation myocardial infarction or cardiogenic shock^32^. To overcome this problem, we constructed the ML risk model using the light GBM with the JCD-KiCS registry to provide the risk of AKI with seven variables comparable to the NCDR-AKI risk model with the 12 variables. Although the more complete risk model would be better with a certain amount of variables, there is a barrier to its adoption in the current practice despite the potential capacity of electronic health records and its real-time automatic risk calculation^33^. In this regard, we created a convenient risk calculator with seven variables in the website, which promotes the adoption into the contemporary medicine. In addition, our study has demonstrated that the risk scores can be validated and shared across different regions by applying the ML based-methods^10^. The study process was possible since JCD-KiCS was developed in line with NCDR CathPCI coding dictionary (version 4.0) and future studies are needed whether the concept can be expanded to other the cardiovascular conditions.

To elucidate the important features among the 12 NCDR variables, we selected them through the Lasso and SHAP methods, which are widely used to select variables^24,34^. We also applied the light GBM which has been developed recently as an ML method, but it generally reduces calculation time and might reduce the burden on the website^15^. It is known that using continuous variables that are suitable for light GBM may enhance predictability in the testing dataset^13^. In addition, it also allows missing values for prediction, which is more advantageous than the conventional logistic regression model. For example, the previous study demonstrated the usefulness of light GBM to build an accurate risk prediction for asthma exacerbation with possessing the advantage of identifying missing values as a unique entity^22^. Importantly, our study is unique in that we created the calculator on the website to assist interventional cardiologists in identifying high-risk patients for AKI after PCI, highlighting the importance of implementing the risk model in physicians.

We analyzed the light GBM with variables without IABP, even though the SHAP or Lasso methods demonstrated that IABP is a pertinent feature since the use of IABP is operator-dependent, and the benefit of IABP is considered to be reduced over the past years^26^. In 2012, IABP-SHOCKIItrial demonstrated IABP did not reduce mortality in patients with CS and following meta-analyses validated the finding^26,27,35,36^. In addition, the observational study also confirmed negative impact of IABP in a variety of clinical settings such as CS, ST-elevation myocardial infarction, heart failure, left main lesions, or three-vessel disease. Given these data, the indications of IABP use became less established during our study period, which extended more than a decade. Therefore, we generated a model without IABP, which is more universal and feasible in current practice than the conventional NCDR-AKI risk model.

Technically, the AUC in the new model was comparable to that of the NCDR-AKI risk model (AUC 0.72)^9^. Better estimation of the AKI incidence before PCI would be beneficial as a foundation for individualized care because it could directly lead to increased use of preventive strategies such as adjusting preprocedural hydration and the amount of contrast volume, deciding to use an intravascular ultrasound and RenalGuard (RenalGuard Solutions, Inc., MA, USA), and management during hospitalization by timely sequential creatinine measurement and follow-up after discharge, which might improve patient outcomes^3,37,38^. In addition, since the incidence of AKI varies among physicians, the showing the AKI risk itself before PCI with simple calculation is useful to reduce the incidence of AKI and its associated mortality and cost^3,39,40^. Moreover, the preprocedural risk of AKI can enhance the patient consent process and risk/benefit evaluation because revascularization for patients with stable coronary artery disease is questionable given the data of recent randomized control trials including the patients with chronic kidney disease^41,42^.

Our study had some limitations. First, we selected our patient cohort from a prospective observational study, which was not initially designed for a focused investigation of the association of AKI after PCI. Second, we excluded patients whose information on AKI after PCI was missing. Although the creatinine levels in relatively stable patients were not consistently assessed, these exclusions could have created a bias in our results. Third, unadjusted confounders are unavoidable given its observational cohort study in nature, however, our registry collaborated with NCDR CathPCI registry and obtained covariates which are known to be confounders. Fourth, although SHAP and Lasso selected important variables, collinearity between the two factors, such as heart failure and cardiogenic shock, cannot be excluded. However, previous reports from the NCDR CathPCI registry used both variables as independent predictors through logistic regression models^9,43,44^. Fifth, our model should be validated using other registries; however, our registry already validated the NCDR registry AKI risk model. Because our registry was constructed in line with the ACC NCDR under mutual collaboration, we were able to use the variable under an identical coding system and conduct direct comparison; therefore, in a way, this study itself is an external validation of the ACC NCDR CA-AKI prediction model using the ML technique^10,11^. Since our registry was constructed in line with NCDR under mutual collaboration, we were able to use the variable under identical coding system and conduct direct comparison; therefore, in a way, this study itself is an external validation of NCDR AKI prediction model, will application of the ML technique. Finally, the different baseline characteristics of the training and testing datasets were shown; however, the incidence of AKI was also different, and we demonstrated a similar AUC between the training and testing datasets despite the different patient characteristics. Herein, we consider that the consistency of the model over different time intervals is a major strength of this study.

Finally, in the JCD-KiCS registry, the data were also extracted from the laboratory values obtained during the entire 30-day follow-up period after the indexed procedure, which may have overestimated the incidence of AKI. However, peak creatinine levels are often observed three to 5 days after contrast exposure^45^, which can be used to estimate the true incidence of AKI.

Notwithstanding the limitations, the AKI risk model after PCI using ML enabled adequate quantification of its risk with fewer clinical variables. Our ML model is meaningful and can replace the conventional NCDR-AKI risk model, given that uses almost half the number of variables. ML techniques may aid in enhancing the international use of validated risk models.

## Supplementary Information

Supplementary Information.

## Abbreviations

AKI: Acute kidney injury
PCI: Percutaneous coronary intervention
NCDR: National cardiovascular data registry
GBM: Gradient boosting model
Lasso: Least absolute shrinkage and selection operator
SHAP: SHapely Additive exPlanations
JCD-KiCS: Japanese cardiovascular database-keio inter-hospital cardiovascular studies
eGFR: Estimated glomerular filtration rate
CS: Cardiogenic shock
CPA: Cardiopulmonary arrest
IABP: Intra-aortic balloon pump
