# Supplementary material for: Machine learning prediction model of acute kidney injury after percutaneous coronary intervention
Source: Sci Rep. 2022 Jan 14;12:749. doi: 10.1038/s41598-021-04372-8 (PMC8760264; doi:10.1038/s41598-021-04372-8)
Supplement: Supplementary file 1 — Supplementary Information. [file 41598_2021_4372_MOESM1_ESM.docx]

Supplemental Table 1: Baseline characteristics of all patients with different study periods

|  | Training dataset  N=16,644 (86.6%) | Testing dataset  N=2,578 (13.4%) | P value | | |
| --- | --- | --- | --- | --- | --- |
| Age | 68.3 (11.3) | 69.5 (11.4) | <0.001 | | |
| Male | 12,421 (79.0) | 1,980 (80.5) | 0.12 | | |
| Creatinine value (mg/dl) | 1.03 ±0.92 | 1.05 ±0.77 | 0.39 | | |
| eGFR (mL/min./1.73 m^2^) | 64.0 ±20.6 | 61.6 ±19.2 | <0.001 | | |
| Baseline Hemoglobin (g/dl) | 13.4 ±2.1 | 13.4 ±2.1 | 0.34 | | |
| Previous myocardial infarction | 3,593 (22.9) | 563 (22.9) | 1.00 | | |
| Previous heart failure | 1,354 ( 8.6) | 275 (11.2) | <0.001 | | |
| Diabetes mellitus | 6,397 (41.0) | 1,073 (43.7) | 0.012 | | |
| Cerebrovascular disease | 1,367 ( 8.7) | 214 ( 8.7) | 1.00 | | |
| Peripheral artery disease | 1,303 ( 8.3) | 205 ( 8.3) | 1.00 | | |
| Chronic lung disease | 500 ( 3.2) | 95 ( 3.9) | 0.093 | | |
| Hypertension | 11,607 (74.1) | 1,799 (73.2) | 0.35 | | |
| Dyslipidemia | 10,275 (65.7) | 1,542 (62.7) | 0.005 | | |
| Previous PCI | 5,636 (35.8) | 1,003 (40.8) | <0.001 | | |
| Previous coronary bypass | 742 ( 4.7) | 123 ( 5.0) | 0.57 | | |
| Heart failure on admission | 1,985 (11.9) | 204 ( 7.9) | <0.001 | | |
| Cardiogenic shock on admission | 774 ( 4.7) | 101 ( 3.9) | 0.11 | | |
| Cardiopulmonary arrest  on admission | 485 ( 2.9) | 80 ( 3.1) | 0.63 | | |
| Radial artery approach | 7,746 (46.7) | 2,141 (83.1) | <0.001 | | |
| Intra-aortic balloon pump | 1,297 ( 7.8) | 128 ( 5.0) | <0.001 | | |
| ST-elevation myocardial infarction | 4,063 (24.5) | 525 (20.4) | <0.001 | | |
| UA/NSTEMI | 3,884 (23.4) | 515 (20.0) | <0.001 | | |
| In-hospital outcomes |  |  |  |  |  |
| In-hospital mortality | 381 ( 2.3) | 39 ( 1.5) | 0.015 |  |  |
| All complications | 1,628 ( 9.8) | 175 ( 6.8) | <0.001 |  |  |
| Coronary Dissection | 161 ( 1.0) | 7 ( 0.3) | 0.001 |  |  |
| Coronary Perforation | 143 ( 0.9) | 10 ( 0.4) | 0.017 |  |  |
| Myocardial infarction | 274 ( 1.6) | 18 ( 0.7) | <0.001 |  |  |
| Cardiogenic shock | 333 ( 2.0) | 31 ( 1.2) | 0.007 |  |  |
| Heart failure | 341 ( 2.0) | 28 ( 1.1) | 0.001 |  |  |
| Cerebral infarction | 73 ( 0.4) | 11 ( 0.4) | 1.00 |  |  |
| Intracranial hemorrhage | 15 ( 0.1) | 4 ( 0.2) | 0.52 | |  |
| Cardiac tamponade | 54 ( 0.3) | 9 ( 0.3) | 0.99 | |  |
| Hemodialysis | 190 ( 1.1) | 20 ( 0.8) | 0.12 | |  |
| Transfusion | 470 ( 2.8) | 68 ( 2.6) | 0.64 | |  |
| Bleeding all | 541 ( 3.3) | 76 ( 2.9) | 0.45 | |  |
| Puncture site bleeding | 152 ( 0.9) | 14 ( 0.5) | 0.076 | |  |
| Puncture site hematoma | 126 ( 0.8) | 12 ( 0.5) | 0.13 | |  |
| Peritoneal bleeding | 24 ( 0.1) | 0 ( 0.0) | 0.10 | |  |
| Gastrointestinal bleeding | 66 ( 0.4) | 8 ( 0.3) | 0.63 | |  |
| Genitourinary bleeding | 22 ( 0.1) | 0 ( 0.0) | 0.13 | |  |
| Other bleeding | 229 ( 1.4) | 41 ( 1.6) | 0.44 | |  |
| Acute kidney injury | 1,587 ( 9.5) | 213 ( 8.3) | 0.043 | |  |

±: standard deviation, eGFR: estimated glomerular filtration rate, PCI: percutaneous coronary intervention UA/NSTEMI: unstable angina/non ST-elevation myocardial infarction
